# Elderly care in daily living in rural Vietnam: Need and its socioeconomic determinants

**DOI:** 10.1186/1471-2318-11-81

**Published:** 2011-12-02

**Authors:** Le V Hoi, Pham Thang, Lars Lindholm

**Affiliations:** 1 Centre for Global Health Research, Department of Public Health and Clinical Medicine, Umeå University, S-901 85, Umeå, Sweden; 2 Institute of Preventive Medicine and Public Health, Hanoi Medical University, 01, Ton That Tung Street, Hanoi City, Vietnam; 3 Central Health Information and Technology Institute, 135, Nui Truc Street, Hanoi City, Vietnam; 4 National Institute of Gerontology, 1A, Phuong Mai Street, Hanoi, Vietnam; 5 Ageing and Living Conditions Programme, Centre for Population Studies, Umeå University, S-901 85, Umeå, Sweden

## Abstract

**Background:**

The proportion of older people is increasing rapidly in Vietnam. The majority of the elderly live in rural areas. Their health status is generally improving but this is less pronounced among the most vulnerable groups. The movement of young people for employment and the impact of other socioeconomic changes leave more elderly on their own and with less family support. This study aims to assess the daily care needs and their socioeconomic determinants among older people in a rural setting.

**Methods:**

In 2007, people aged 60 years and older, living in 2,240 households, were randomly selected from the *FilaBavi Demographic Surveillance System *(DSS). They were interviewed using structured questionnaires to assess needed support in activities of daily living (ADLs). Individuals were interviewed about the presence of chronic illnesses that had been diagnosed by a physician. Participant socioeconomic characteristics were extracted from the *FilaBavi *repeat census. The repeat census used a repeat of the same survey methods and questions as the original FilaBavi DSS. Distributions of study participants by socioeconomic group, supports needed, levels of support received, types of caregivers, and the ADL index were described. Multivariate analyses were performed to identify socioeconomic determinants of the ADL index.

**Results:**

The majority of older people do not need of support for each specific ADL item. Dependence in instrumental or intellectual ADLs was more common than for basic ADLs. People who need total help were less common than those who need some help in most ADLs. Over three-fifths of those who need help receive enough support in all ADL dimensions. Children and grandchildren are the main caregivers. Age group, sex, educational level, marital status, household membership, working status, household size, living arrangement, residential area, household wealth, poverty status, and chronic illnesses were determinants of daily care needs in old age.

**Conclusions:**

Although majority of older people who needed help received enough support in daily care, the need of care is more demanded in disadvantaged groups. Future community-based, long-term elderly care should focus on instrumental and intellectual ADLs among the general population of older people, and on basic ADLs among those with chronic illnesses. Socioeconomic determinants of care needs should be addressed in future interventions.

## Background

A rapid aging trend, with declines in fertility and mortality, was observed in Vietnam during recent decades [1]. The proportion of people 60 years and older increased from 6.7% in 1979 to 9.2% in 2006 [2]. This proportion is projected to be 26.1% by 2050 [3]. Vietnamese life expectancy at birth increased from 66 years in 1990 to 72 years in 2006 [4,5], and is projected to increase to 80.3 years by 2050 [3]. There is evidence that life expectancy in rural areas generally increases at old age, except among the most vulnerable groups such as people living below the poverty line and those living without adult children. In the later groups, life expectancy decreased [1].

In 2007, the total population of Vietnam reached 85 million, with 72.6% of residents living in rural areas [6]. People aged 60 years and over are considered elderly or older people. The majority (72.9%) live in rural areas [7] with more disadvantaged living conditions [8,9]. Older people, especially in rural areas, are more likely to rely on domestic sources of economic support than on the social security system [10]. There is an increasing trend of temporary migration from rural to urban areas among the young labour force because of better employment opportunities [11]. This leaves more older people living on their own with less physical and emotional support from family members [12].

Although greater life expectancy at old age is an indicator of successful ageing [13], it also means that more older people suffer from non-communicable diseases (NCDs) [14]. The most common NCDs among elderly Vietnamese are cardiovascular disease, diabetes, kidney disease, and cancer [15]. The elderly also suffer from accidents, frequent illnesses, and multiple, concurrent health disorders. A survey found that 60% of older people were ill during the prior four weeks and 70% suffer from NCDs [15]. The proportion of older people with self-reported poor health increases with age, from 50% at age 65-74 years to 81% at age 85 and above [16].

Vietnamese household health expenditures predominantly consist of out-of-pocket payments and accounted for 67% of expenses in 2005 [17]. Disparities in health and health care are widening between socioeconomic groups, as well as between rural and urban areas. In particular, the rural elderly have less access to health care than those in urban areas [16]. Access to health services among older people is often limited by mobility problems and the inability to afford health care services, especially long-term care [15]. Care for the elderly is a major need and a prioritized issue in health and social policies.

The relations between greater need of care and increased number of diseases and functional impairments are well known [18]. Assessment of the functional capacity of older people in activities of daily living (ADLs) is widely used in clinical studies and community-based surveys [19]. Disabilities in ADLs are not only predictors of institutionalization, hospitalization and mortality, but also important indicators of the need for formal and informal community and home care [20].

Elderly health outcomes are generally considered to be influenced by genetic factors at the molecular level, social and behavioural factors at the individual level, socioeconomic and physical environmental factors at the community level, as well as complex interactions among all of these [21]. Socioeconomic determinants of ADLs, such as age group, sex, educational level, income, expenditure, living conditions, living arrangement, regions, and chronic diseases are well documented in different settings of other countries [22-25].

To date, elderly health care in Vietnam mainly relies on daily support of family caregivers and short-term care in health facilities. However, little is known about elderly daily care needs in Vietnam's new context of multi-dimensional transitions. This is particularly true in the rural setting. Therefore, in order to provide evidence for designing appropriate national health and social policies for elderly care, this study aims to assess daily care needs and their socioeconomic determinants among older people in a rural setting.

## Methods

### Study setting and the FilaBavi surveillance system

The study was conducted as a part of a larger study in 2007. We previously analysed remaining life expectancy [1], health-related quality of life [26], and attitudes and willingness to pay for formal care [27]. Data were collected within the longitudinal demographic and health surveillance system of *FilaBavi *[28]. This field site operates in the rural Bavi District of Vietnam, and covers an area of 410 km^2^. Bavi District has lowland, highland and mountainous areas, of which 30% is used for agriculture and 17% is forest. The population was 262,763 in 2007. Among adults over age 20, the majority completed primary/secondary school (65% of men, 72% of women) or high school/higher education (34% of men, 23% of women) with the rest being illiterate. Approximately two-thirds of the population are farmers (39% of men, 57% of women) or other workers (31% of men, 9% of women). The remainder are business people, students, government staff, retired persons or others.

The *FilaBavi *surveillance system consists of a representative sample of 67 of 352 district clusters, randomly selected since 1999 with a probability proportional to population size in each cluster. A cluster is defined as an administrative unit, usually a village. If a village is too large, it may be divided into two clusters. On average, there are 600-700 inhabitants in each cluster. Initially, 11,089 households and 51,024 inhabitants were included for surveillance. In 2007, 53,927 individuals were followed up by *FilaBavi*, accounting for approximately 20.5% of the total district population. People aged 60 and over represented 11.5% of the total follow-up population at the 2007 mid-year point.

### Study design, sampling and sample size

A household cross-sectional survey was conducted. Sample size was calculated based on estimating a proportion in a population-based survey. Using an estimated proportion of 13% of older people who need support for daily living in rural Vietnam [29], and an estimation error of 2.6%, a sample size of 2,699 older people was required. This adjusts for a design effect of two for cluster sampling of *FilaBavi*. This was then doubled to increase the robustness of the multivariate analysis, and allows a 10% non-response rate. This figure is approximately equal to 50% of all people aged 60 or older in the *FilaBavi *sampling frame.

Subsequently, 50% of households with older people were randomly selected for a household cross-sectional survey. Face-to-face interviews were conducted with all the older people and their family representatives in the selected households. This included 2,255 households with 2,968 people. During the survey period of July to October 2007, 166 households were excluded due to absence of the older people. Each of these cases was replaced with the nearest unselected household with older people. In total, 2,240 households with 2,873 older people were included in the study.

### Variable measurement and data collection

Data on household economic status, including land area, structural housing components, assets, sanitary conditions, income, expenditures, and debt, were extracted from the mid-2007 *FilaBavi *repeat census dataset. Assets were classified according to certain categories, such as furniture, communication and electrical equipment, type of vehicle, agricultural machines, cattle, and others. These items were classified as "present" or "not present", regardless of their quantity or quality. Sanitary conditions were assessed as sources of water for drinking and cooking, type of latrine, and presence of a bathroom. All types of income (agriculture, breeding, forestry and others) were recorded to provide the total income of a given household. The sum of daily food expenditure was multiplied by 30 days and added to the sum of other monthly expenditures to estimate total monthly household expenditure. Monthly income and expenditures were then divided by household size to generate "per capita" variables. Geographical areas where the households are located (ie, lowlands, highlands, mountainous areas) were extracted together with household economic data.

Using structured questionnaires, face-to-face interviews were performed at the houses by 52 trained *FilaBavi *field personnel. These included questions on support needed in ADLs, and individual and household characteristics of the elderly, such as date of birth, sex, education, marital status, household head status, living arrangements, work status (eg, working in own rice fields or not working), and the presence of chronic illnesses. Three scales of ADLs were applied when measuring the daily care needs, including Katz's basic ADLs [30] (bathing, dressing, toilet use, transferring in and out of bed or chair, urine and bowel continence, and eating), instrumental ADLs (cleaning house, cooking, shopping, travelling) and intellectual ADLs (writing, reading, listening to the radio, watching TV). All the items of these scales are essential ADLs. Support needs for each activity (no, some need, complete dependence) were assessed, together with levels of support received (none, not enough, enough) and caregiver types (eg, son/daughter, grandchild, relatives). The wording of the questions about these activities was made suitable to the context of rural Vietnam.

Respondents were asked about the presence of hypertension, diabetes, arthritis/osteoarthritis or rheumatism, stroke, angina or angina pectoris, bronchitis or emphysema/chronic obstructive pulmonary disease, depression, cancer, cataracts, and missing teeth. Only chronic illnesses that were reported as "diagnosed by a physician" were recorded. The number of chronic illnesses for each participant was classified into four categories (no disease, one disease, two diseases, or three or more diseases).

Six field supervisors reviewed each completed questionnaire and randomly selected 5% for re-interview. Each questionnaire with missing or irrelevant values was returned to the field personnel for checking and completion following re-visit to the corresponding household. Double data-entry using Epi-Data 3.1 was performed to check for inconsistent values for each variable, and correction of data-entry errors was based on the actual data from the completed questionnaires.

### Statistical analysis

Datasets from the present survey and the repeat census were linked and analysed using STATA 10. An indicator of household long-term economic conditions, the household wealth index, was calculated as the first component for all economic variables from the repeat census. Classification of household wealth quintile was based on the hierarchy among all *FilaBavi *households. Household poverty status was classified using the national poverty line for rural areas, and based on monthly per capita income equal to VND 200,000 (USD 12.50) for 2006-2010 [31]. The poverty status reflects the household short-term economic conditions.

An index was calculated for each ADL scale by summing up the score from each activity (score is 0 if no need or some need; score is 1 if complete dependence). The basic ADL index ranges from 0 to 6. The instrumental and intellectual indices range from 0 to 4. Distribution of study participants by socioeconomic group, need of any support for each activity (no need vs. some need or complete dependence), level of support received among those in need, types of caregivers among those who receive support, and ADL indices were described using percentages and corresponding 95% confidence intervals.

Multivariate analyses using Poisson regression were performed to measure the effect of socioeconomic factors on an ADL index. Being female, aged 80 and above, illiterate, widowed, living without spouse or other family members, position as household member, not working in old age, belonging to the poorest quintile, smaller household size, residence in mountainous areas, and living above the national poverty line were the reference categories. A backward stepwise procedure with a p-value of 5% for removal was used to identify significant factors to remain in the final multivariate model. Robust standard errors were used for accurate estimation of the model cluster data parameters [32].

### Ethical considerations

Ethical approval for the demographic surveillance system of *FilaBavi*, including data collection on socioeconomic statistics, was given by the Research Ethics Committee at Umeå University, Sweden (reference number 02-420). The present study was also approved by the Research Ethics Committee at Hanoi Medical University (reference number 51/HMU-RB).

## Results

The socioeconomic characteristics of the study participants are summarized in Additional file 1, Table S1. These are described in detail elsewhere, together with the general health status of the participants [1,26]. There is a predominance of older people who are female, 65-74 years of age, literate, married or widowed, living without a spouse, living with children or grandchildren, belonging to households in the middle to richest wealth quintiles, and living above the national poverty line. Just over half of the household heads and two-fifths of the older people are still working. Approximately 10% of the older people live alone. In addition, just over two-fifths of the elderly suffer from at least one chronic illness.

Distributions of older people by ADL indices for different age groups are presented in Table 1. Instrumental and intellectual ADLs are the most frequent problems, and the frequencies increase with age. The proportion of people who are independent in basic ADLs (index = 0) drops from 97.6% among those over 64 years of age, to 86.7% among those above 84 years. The proportion decreases significantly with each increment in ten-year age interval. Dependency in the other ADL scales is more frequent. Almost three-fifths of the elderly have problems with one or more ADL in each scale, and the proportion increases by five year intervals. Most people are dependent in 1-2 instrumental or intellectual ADLs, except those aged 80 and above for whom dependency in 3-4 items is common.

**Table 1:** Distributions of older people by activities of daily living (ADLs) at different ages

	*Basic ADLs*		*Instrumental ADLs*		*Intellectual ADLs*	
	
Age/Index	%	95%CI	%	95%CI	%	95%CI
*Age 60+ yr*						
0	97.6	97.0 - 98.1	32.3	30.6 - 34.1	42.6	40.8 - 44.4
1-2	0.8	0.5 - 1.0	47.9	46.1 - 49.7	44.1	42.3 - 46.0
3-4	0.7	0.4 - 1.0	19.8	18.3 - 21.2	13.3	12.0 - 14.5
5-6	1.0	0.6 - 1.3	-	-	-	-

*Age 65+ yr*						
0	97.0	96.3 - 97.7	26.4	24.6 - 28.2	35.9	33.9 - 37.8
1-2	0.9	0.5 - 1.3	50.0	47.9 - 52.0	48.0	46.0 - 50.1
3-4	0.8	0.5 - 1.2	23.6	21.9 - 25.4	16.1	14.6 - 17.7
5-6	1.2	0.8 - 1.7	-	-	-	-

*Age 70+ yr*						
0	96.2	95.3 - 97.1	20.2	18.3 - 22.2	27.3	25.1 - 29.4
1-2	1.2	0.7 - 1.7	50.6	48.2 - 53.0	53.0	50.6 - 55.4
3-4	1.1	0.6 - 1.7	29.2	27.0 - 31.4	19.8	17.9 - 21.7
5-6	1.4	0.9 - 2.0	-	-	-	-

*Age 75+ yr*						
0	94.9	93.6 - 96.2	14.2	12.2 - 16.3	19.1	16.8 - 21.5
1-2	1.5	0.7 - 2.2	48.2	45.2 - 51.1	55.7	52.7 - 58.6
3-4	1.7	1.0 - 2.5	37.6	34.7 - 40.5	25.2	22.6 - 27.8
5-6	1.9	1.1 - 2.7	-	-	-	-

*Age 80+ yr*						
0	92.2	90.0 - 94.4	7.1	5.1 - 9.2	12.6	9.9 - 15.3
1-2	2.2	1.0 - 3.4	43.0	39.0 - 47.0	54.3	50.3 - 58.4
3-4	2.4	1.1 - 3.6	49.9	45.9 - 54.0	33.1	29.3 - 36.9
5-6	3.2	1.8 - 4.7	-	-	-	-

*Age 85+ yr*						
0	86.7	82.6 - 90.8	3.4	1.2 - 5.6	7.6	4.4 - 10.8
1-2	4.2	1.8 - 6.6	33.8	28.1 - 39.6	47.1	41.1 - 53.2
3-4	3.8	1.5 - 6.1	62.7	56.9 - 68.6	45.2	39.2 - 51.3
5-6	5.3	2.6 - 8.1	-	-	-	-

Needed support for specific items in each ADL scale among people aged 60 and older is described in Table 2. The majority of respondents have "no need". The proportion of older people that need help is highest for intellectual ADLs (13-32%), followed by instrumental ADLs (3-13%), and basic ADLs (3-8%). Few older people belong to the category "complete dependence". Reading and writing are the most common problems in intellectual ADLs. The most common problem in instrumental ADLs is travelling and the most common in basic ADLs is bathing.

**Table 2:** Distributions of people aged 60 years and older by support needs for specific activities of daily living (ADLs)

	Sample size	No need		Some need		Complete dependence	
	
Type of ADL		*%*	*95%CI*	*%*	*95%CI*	*%*	*95%CI*
*Basic ADLs*							
Bathing	2,873	92.5	91.6 - 93.5	5.3	4.5 - 6.1	2.2	1.7 - 2.7
Dressing	2,873	95.5	94.8 - 96.3	2.5	2.0 - 3.1	2.0	1.4 - 2.5
Toilet use	2,873	96.6	95.9 - 97.2	2.6	2.0 - 3.2	0.8	0.5 - 1.2
Transferring	2,873	95.6	94.8 - 96.3	3.1	2.5 - 3.8	1.3	0.9 - 1.7
Urine/bowel continence	2,873	95.3	94.5 - 96.1	3.0	2.3 - 3.6	1.7	1.3 - 2.2
Eating	2,873	96.2	95.5 - 96.9	2.6	2.0 - 3.2	1.2	0.8 - 1.6

*Instrumental ADLs*							
Cleaning house	2,373	97.2	96.6 - 97.9	2.7	2.0 - 3.4	0.1	-0.03 - 0.2
Cooking	2,063	96.4	95.6 - 97.2	3.5	2.7 - 4.3	0.1	-0.03 - 0.2
Shopping	1,229	96.6	95.6 - 97.6	3.3	2.3 - 4.3	0.2	-0.06 - 0.4
Travelling	2,047	86.8	85.3 - 88.2	10.0	8.7 - 11.3	3.2	2.5 - 4.0

*Intellectual ADLs*							
Writing	1,398	71.8	69.5 - 74.2	21.9	19.7 - 24.1	6.3	5.0 - 7.6
Reading	1,651	67.9	65.6 - 70.1	23.9	21.9 - 26.0	8.2	6.9 - 9.5
Listening to the radio	2,651	86.8	85.6 - 88.1	9.7	8.6 - 10.8	3.5	2.8 - 4.2
Watching TV	2,623	83.4	82.0 - 84.8	12.3	11.1 - 13.5	4.3	3.5 - 5.1

ADL supports received among those in need are presented in Table 3. A significant group does not receive any or enough help. This is most pronounced for instrumental and intellectual ADLs. More than 70% of individuals have enough support in all ADL dimensions except shopping (62.5%). There is a wide range in proportions of those not receiving enough support for different items. This varied from 5-14% for basic ADLs, 9-29% for intellectual ADLs, and 20-38% for instrumental ADLs with the exception of only 4% for travelling. The sample sizes of those who need help in specific ADL types varied from around 80 to 600. This affects the confidence intervals around percentages of those who received no or not enough support.

**Table 3:** Distributions of people aged 60 years and older by support received for specific activities of daily living (ADLs)

	No support		Not enough support		Enough support	
	
Type of ADL	*%*	*95%CI*	*%*	*95%CI*	*%*	*95%CI*
*Basic ADLs*						
Bathing	1.4	-0.2 - 3.0	9.8	5.8 - 13.8	88.8	84.6 - 93.1
Dressing	3.1	0.1 - 6.2	8.6	3.7 - 13.5	88.3	82.6 - 93.9
Toilet use	0	0	5.1	0.1 - 9.5	94.9	90.5 - 99.3
Transferring	0	0	14.1	8.0 - 20.2	85.9	79.8 - 92.0
Urine/bowel continence	1.5	-0.1 - 3.6	12.8	7.0 - 18.5	85.7	79.7 - 91.7
Eating	0	0	13.9	7.3 - 20.5	86.1	79.5 - 92.7

*Instrumental ADLs*						
Cleaning house	1.5	-1.5 - 4.6	27.7	16.5 - 38.9	70.7	59.4 - 82.1
Cooking	0	0	20.5	11.1 - 30.0	79.5	70.0 - 89.0
Shopping	0	0	37.5	21.8 - 53.2	62.5	46.8 - 78.2
Travelling	0	0	4.4	2.0 - 6.9	95.6	93.1 - 98.0

*Intellectual ADLs*						
Writing	0.5	-0.2 - 1.2	9.3	6.4 - 12.2	90.2	87.2 - 93.1
Reading	1.0	0.1 - 1.8	13.7	10.7 - 16.7	85.3	82.3 - 88.4
Listening to the radio	0.3	-0.3 - 0.9	29.3	24.4 - 34.2	70.4	65.5 - 75.3
Watching TV	0.2	-0.2 - 0.7	24.1	20.1 - 28.1	75.7	71.7 - 79.7

Caregivers for the different ADL dimensions are described in Table 4. Children and grandchildren are the main ADL caregivers while spouses are not as important. Children are the most frequent ADL caregivers for basic ADLs and travelling. Children and grandchildren play the same role in the other instrumental and intellectual activities. Support from spouses is received by only 9-12% of those who require assistance with basic ADLs. Less than 7% receive spousal support for instrumental ADLs and 5% for intellectual ADLs. In addition, other caregivers, including relatives and neighbours, provide the care measured by most ADL scales for less than 3.5% of older people; this excludes 26.8% from neighbours, 7.3% from relatives for shopping, and 4.9% from relatives for travelling.

**Table 4:** Distributions of people aged 60 years and older by main caregivers for specific activities of daily living (ADLs)

		Son/daughter		Grandchild		Spouse	
		
Type of ADL	Sample size	*%*	*95%CI*	*%*	*95%CI*	*%*	*95%CI*
*Basic ADLs*							
Bathing	218	80.7	75.5 - 86.0	59.6	53.0 - 66.2	11.9	7.6 - 16.3
Dressing	129	80.6	73.7 - 87.5	56.6	47.9 - 65.3	12.4	6.6 - 18.2
Toilet use	101	87.1	80.5 - 93.8	64.4	54.9 - 73.9	11.9	5.5 - 18.3
Transferring	130	74.6	67.0 - 82.2	54.6	45.9 - 63.3	9.2	4.2 - 14.3
Urine/bowel continence	134	87.3	81.6 - 93.0	60.5	52.1 - 68.8	9.7	4.6 - 14.8
Eating	108	80.6	73.0 - 88.1	49.1	39.5 - 58.7	11.1	5.1 - 17.1

*Instrumental ADLs*							
Cleaning house	67	74.6	63.9 - 85.3	71.6	60.6 - 82.7	4.5	-0.6 - 9.6
Cooking	75	74.7	64.6 - 84.7	80.0	70.7 - 89.3	6.7	0.9 - 12.5
Shopping	42	57.1	41.5 - 72.8	54.8	39.1 - 70.5	0	0
Travelling	268	96.3	94.0 - 98.6	71.6	66.2 - 77.1	0.8	-0.3 - 1.8

*Intellectual ADLs*							
Writing	391	32.1	27.4 - 36.7	25.6	21.2 - 29.9	1.5	0.3 - 2.8
Reading	528	36.7	32.6 - 40.9	37.5	33.4 - 41.6	1.0	0.1 - 1.8
Listening to the radio	347	84.7	80.9 - 88.5	86.5	82.8 - 90.1	3.5	1.5 - 5.4
Watching TV	438	79.7	75.9 - 83.5	82.2	78.6 - 85.8	4.4	2.4 - 6.3

Distributions of older people who need any support, either some or complete support, for at least one item in each ADL scale by socioeconomic group are presented in Additional file 1, Table S2. Almost ten per cent reported a need of some support in a basic ADL while over two-thirds were in need of some support in an instrumental or intellectual ADL. Percentages of people who need any support increased at older age groups. There are specific gaps in support needs by sex, educational level, marital status, living arrangement, household head status, working status, household size, area of residence, and household economic conditions.

Table 5 presents the simultaneous relationship of socioeconomic factors with ADL indices. Younger age groups, literacy, married status, living alone, position as household head, working until old age, smaller household size, living in the highlands or lowlands, and belonging to better wealth quintiles are indicators of having fewer ADLs that are completely dependent on caregiver support. Being separated/divorced/single, living with child/grandchild, living under the national poverty line, and having a higher number of chronic illnesses are risk factors of having a higher number of complete dependence in ADLs. Being male is an indicator of having a higher number of instrumental ADLs but its effect is the opposite for intellectual ADLs. A contrasting picture is observed for instrumental and intellectual ADLs among those living with a spouse.

**Table 5:** Effect of socioeconomic factors and number of noncommunicable diseases (NCDs) on elderly activities of daily living (ADLs) for older people

Socioeconomic factors/Terms	Basic ADLs			Instrumental ADLs			Intellectual ADLs		
	
	*Coef*.	*IRR*	*P-value*	*Coef*.	*IRR*	*P-value*	*Coef*.	*IRR*	*P-value*
Aged 60-69 yr	-1.382	0.25	0.002	-0.712	0.49	< 0.001	-0.415	0.66	< 0.001
Aged 70-79 yr	-1.385	0.25	< 0.001	-0.366	0.69	< 0.001	-0.160	0.85	< 0.001
Male				0.424	1.53	< 0.001	-0.378	0.69	< 0.001
High school & above education				-0.459	0.63	< 0.001	-1.990	0.14	< 0.001
Primary/secondary school education				-0.347	0.71	< 0.001	-0.810	0.45	< 0.001
Able to read and write				-0.248	0.78	< 0.001	-0.284	0.75	< 0.001
Married							-0.176	0.84	< 0.001
Separated/divorced/single				0.303	1.35	0.022			
Living with spouse				-0.118	0.89	0.006	0.116	1.12	0.041
Living with son/daughter				0.209	1.23	0.001			
Living with grandchild				0.152	1.16	0.002	0.083	1.09	0.026
Living alone	-2.690	0.07	0.010	-0.452	0.64	< 0.001			
Household head	-0.911	0.40	0.013				-0.251	0.78	< 0.001
Working or employed	-2.537	0.08	0.003	-0.530	0.59	< 0.001	-0.175	0.84	< 0.001
Household size ≤4				-0.117	0.89	0.002			
Lowland area				-0.234	0.79	< 0.001			
Highland area				-0.178	0.84	< 0.001	-0.088	0.92	0.003
Living below NPL				0.094	1.10	0.012	0.090	1.10	0.019
Richest quintile							-0.413	0.66	< 0.001
Richer quintile							-0.297	0.74	< 0.001
Middle quintile							-0.215	0.81	< 0.001
Poorer quintile							-0.175	0.84	< 0.001
Number of NCDs	0.634	1.88	< 0.001	0.067	1.07	< 0.001	0.074	1.08	< 0.001
Constant	-1.400		< 0.001	0.857		< 0.001	1.295		< 0.001
Pseudo R**^2^**	0.245			0.153			0.200		

Coef. = coefficient; IRR = incident rate ratio; NPL = national poverty level; pseudo R^2 ^= pseudo-coefficient of determination

## Discussion

### Needs of daily care at old ages

Functional disability is most often measured in clinical studies and community-based surveys among elderly people by using ADLs [19]. The measurements use different scales and words to assess for difficulties faced or need of support in performing activities. To the best of our knowledge, there are not any published, comparative figures on the daily care needs for older people assessed with the same ADL scales from previous studies in Vietnam. The finding that the majority of older people have no need of support in specific ADL items may be explained by the fact they are mainly at young-old (61.8% at 60-74 years) and middle-old (29.0% at 75-84 years ages.

This study's findings on basic and instrumental ADL indices at age 65 or older are consistent with those documented in the literature. The proportions of basic ADL dysfunction among the community's elderly population are 2-8%, and the elderly are more likely to be dependent in instrumental ADLs than basic ADLs [33]. Complete dependence in both instrumental and intellectual ADLs is much higher and increases by age faster than for basic ADLs. This suggests that future interventions to improve daily care for rural elderly should focus on instrumental and intellectual functions, rather than on basic ADLs.

Among those who express the need for support, most elderly require some help. The proportion of people who do not receive enough support in many instrumental and intellectual ADLs is much higher than for basic ADLs. Furthermore, there is a strong, existing tradition in rural Vietnam that older people live with their children. These characteristics indicate that an improvement in home-based care is more important in future interventions than improving institutional care.

There is an unmet need for support in ADLs while the main sources of support for elderly care are from children and grandchildren. This indicates a discernible need of complementary daily care patterns outside of the family. In the context of an increasing temporary migration of the young labour force from rural to urban areas [11], and the transition from extended households to nuclear households [12], this is especially important. The need for development of a social network and services for community-based long-term elderly care, currently lacking in rural Vietnam, is essential. Social organizations and community members at the grassroots level, together with health professionals in the current health system, should be motivated to participate in the network in order to fill the gaps of formal and informal care.

The proportion of people at different old ages (ie, ≥60, ≥65, ≥70 and ≥75) who need support for at least one basic ADL (ie, 9.6%, 11.7%, 14.5% and 18.8%; Additional file 1, Table S2) are all lower than among rural elderly in Malaysia (14.3%, 19.9%, 25.3% and 32.9%, respectively) [34]. Only at age ≥60 is this proportion lower than among rural elderly in Bangladesh (21.7% for men, 36% for women) [35]. At age ≥65, the proportion is lower than among elderly in the United States (18.4%) [36]. At age ≥80, the proportion (26.2%) is higher than among rural elderly in China (14.3% for men, 18.5% for women) [37]. Explanation of the differences between countries is difficult because of differences in ADL measurement, elderly perspectives and expectations of caregiver supports, and availability and accessibility of social care service networks.

### Demographic determinants of care

Younger-old ages are indicators of a lower index for each ADL scale. This can be explained by better general health status at these younger ages. People with higher educational levels have lower instrumental and intellectual ADL indices. This is likely due to the better health status that results from greater income during working ages [38] as well as greater acquisition of life skills, knowledge and health care [39].

Men have higher indices for basic and instrumental ADLs while women only have a higher intellectual ADL index. This might be due to characteristics associated with patrilineality and patrilocality [40] which still strongly influence rural areas, and the fact that literacy is lower among women. The first aspect may increase the emotional expectation among men of help with basic and instrumental ADLs. Lower literacy among women might lead to a greater need for help with intellectual ADLs.

Married status is a predictor of a lower intellectual ADL index. This may suggest that emotional exchange within a married couple is important in maintaining physical functions at old ages. Another factor may be that married people in rural areas are more likely to be younger than those who are widowed. Thus, being married may correlate with younger age and, as expected, those of younger age are in better health than those who are older. Those who are separated, divorced or single are at risk for a higher instrumental ADL index compared to widowed people in old age.

Previous studies indicate that older people who continue to work have better general health than their counterparts [1,26]. The majority of working people are younger than those who are not working. In turn, working helps people stay healthy. These characteristics may be explained by our finding that working at old age is a positive predictor of a lower ADL index and a lower need for support. Household heads have lower basic and intellectual ADL indices and fewer support needs. This may be because these older people are more likely to have a better health status and higher literacy levels than other household members.

### Living arrangements and need of care

People who live in smaller households have a lower instrumental ADL index. This could result from a lower expectation of having help among those who live in families with fewer potential caregivers, or with a lack of outside caregivers. Living with a spouse increases the likelihood of having a higher intellectual ADL index but a lower instrumental ADL index. This might be because it is easier for pairs of older people to support for each other in intellectual ADLs since instrumental ADLs are physically heavier and are more difficult to share. In rural areas, it is common for older married couples to live temporarily in different households of their children. This phenomenon accounts for 24.3% of married participants in the present study (Additional file 1, Table S1). On one hand, this allows adult children to share responsibilities of nurturing and caring for older parents, especially among the poor. On the other hand, a proportion of older people provide help for their adult children by doing housework or taking care of their grandchildren. This type of living arrangement may also explain the low proportions of having support from a spouse.

That loneliness is a promoting factor for a lower ADL index in both basic and instrumental scales, while living with children or grandchildren is a risk factor for a higher ADL index in one or both of the instrumental and intellectual scales, was surprising. The presence of children and grandchildren may encourage a higher expectation of having ADL support among older people who live with their descendants, while lack of a social network may limit the expectation of outside support among those who live alone.

Living in the lowlands or highlands predicts a lower instrumental ADL index than living in mountainous areas. This could be because people in mountainous areas have a poorer general health status due to more disadvantages in living and health care conditions [8,9,16]. People in the highlands have a lower intellectual ADL index than those in mountainous areas. This may be explained by better health status and higher literacy but more need and conditions for intellectual exchanges (eg, newspapers, TV, radio) among those in the highlands.

### Economic determinants of care

Living below the national poverty line is indicator of higher intellectual and instrumental ADL indices. This economic condition might relate to the ADL indices through different mechanisms. A better economic condition might either contribute to better health status [1] or increase the availability of potential family caregivers who are able to relieve the economic burden.

Higher household wealth promotes a lower intellectual ADL index but does not improve other scales. Household wealth primarily reflects household living conditions and is sensitive to intellectual exchanges. Generally, poverty is more extensively related to ADL scales than wealth.

### A need for long-term care for elderly with chronic illnesses

Variation in the number of chronic illnesses is related to all ADL indices. This is consistent with the finding of a previous study among Canadian seniors [41] where having more than one chronic condition increases the likelihood of dependency. In the current study, the relationship is profound on the basic scale but slight on the other scales. Therefore, older people with chronic illnesses should be targeted with supportive care that focuses on basic ADLs rather than instrumental and intellectual ADLs. This also indicates the need of long-term care for such people because basic ADLs include basic personal care. As chronic illness is prevalent in a large portion of older people (42%), support for long-term elderly care becomes an issue in rural areas.

### Roles of determinants and the most disadvantaged groups

Among many examined socioeconomic factors, only four (ie, age group, loneliness, household head status, working status) significantly relate to basic ADL index. Many more factors relate to the instrumental and intellectual scales. The multivariate model coefficient of determinants of basic ADLs (24.5%) is higher or nearly equal to those for the instrumental (15.3%) and intellectual (20.0%) scales. This suggests that future interventions to improve daily care for the elderly should address socioeconomic determinants of instrumental and intellectual care over those for basic care. This is not to say that factors related to basic care are less important than other aspects of care.

Among older people, approximately 30% are 80 years or older, and 18% are illiterate (Additional file 1, Table S2). These people have the highest number of dependent ADLs. People who are separated/divorced/single, household members, not working, living with children or grandchildren, belonging to the poorest quintiles, or living below the national poverty line are more likely to have a higher ADL index than their counterparts. These disadvantaged groups should be targeted in future interventions to improve daily care for older people.

### Methodological issues

ADLs do not cover all disability domains. Therefore, assessment of elderly care needs may underestimate other care needs for functional impairments that were not assessed. Also, household economic data, particularly expenditures and income, were extracted from repeat census data. These figures fluctuate, and this is especially likely to occur by seasons in rural Vietnam.

The proportion of older people who are illiterate or have low literacy is high in rural areas. People with low literacy may understand the survey questions differently from those who are literate. The majority of illiterate participants need support from literate people when they want to read or write. Some of them might not need any support in writing (19.4%) or reading (10.7%) as they do not have a need for reading or writing, or do not want to communicate by reading or writing.

Radio and television are now popular in rural areas, but not all households have these appliances. People in a rural community live close together. Members of a household usually visit other households. It is the norm in rural areas for those in households without a radio or television to listen to the radio or watch television in the houses of their neighbours or relatives. Survey questions on the need of support in listening to radio or watching television were difficult to respond to among those with mobility disabilities who live in households without such audio-visual equipment.

Because this is a cross-sectional survey, inferential explanations of causal pathways between daily care needs and their determinants cannot be made. Finally, the presence of chronic illnesses might be underestimated because only those diagnosed by physicians were recorded.

## Conclusions

Although the majority of older people who need help receive enough support in daily care, the need of care is greater in disadvantaged groups. Adult children and grandchildren are the main sources of support for the elderly. Care needs are related to age group, sex, educational level, marital status, household membership, working status, household size, living arrangement, residential area, household wealth, poverty status, and presence of chronic illness. Further research to explain these relationships more clearly will be useful.

Development of a social network for improving community-based, long-term elderly care is increasingly necessary. The network should focus on instrumental and intellectual ADLs among the general population of older people, and on basic ADLs among those with chronic illnesses. Home-based care is more important than institutional care for future interventions. Those who are the oldest, illiterate, separated/divorced/single, household members rather than household heads, not working, living with children or grandchildren, belonging to the poorest quintile, living below the national poverty line, and with chronic illnesses should be targeted for daily care in old age.

## List of abbreviations

ADL: Activity of daily living; DSS: Demographic Surveillance Site; NCD: Non-communicable disease; USD: United States dollar; VND: Viet Nam dong.

## Competing interests

The authors declare that they have no competing interests.

## Authors' contributions

LVH conceived and designed the research, developed the tools, supervised data collection, analysed data and drafted the manuscript. LL assisted during the conception phase, advised on the research design and data analyses, and revised the manuscript. PT advised on data analyses and revised the manuscript. Each author read and approved the final manuscript.

## Pre-publication history

The pre-publication history for this paper can be accessed here:

http://www.biomedcentral.com/1471-2318/11/81/prepub

## Supplementary Material

Additional file 1 **Appendix**. Additional information on older people. Click here for file
