# Supplementary material for: Elderly care in daily living in rural Vietnam: Need and its socioeconomic determinants
Source: BMC Geriatr. 2011 Dec 2;11:81. doi: 10.1186/1471-2318-11-81 (PMC3239225; doi:10.1186/1471-2318-11-81)
Supplement: Additional file 1 — Appendix. Additional information on older people. [file 1471-2318-11-81-S1.DOC]

**Appendix:**

**Table S1 – Distributions of older people by socioeconomic group and number of non-communicable diseases (NCDs)**

| Variables | n | % | 95%CI |
| --- | --- | --- | --- |
| *Age (yr)* |  |  |  |
| 60+ | 2,873 | 100.0 | - |
| 65+ | 2,275 | 79.2 | 77.7 – 80.7 |
| 70+ | 1,665 | 58.0 | 56.2 – 59.8 |
| 75+ | 1,096 | 38.2 | 36.4 – 39.9 |
| 80+ | 589 | 20.5 | 19.0 – 22.0 |
| 85+ | 263 | 9.2 | 8.1 – 10.2 |
| *Age group (yr)* |  |  |  |
| 60–69 | 1,208 | 42.0 | 40.2 – 43.9 |
| 70–79 | 1,076 | 37.4 | 35.7 – 39.2 |
| 80–89 | 513 | 17.9 | 16.5 – 19.3 |
| 90+ | 76 | 2.7 | 2.1 – 3.2 |
| *Sex* |  |  |  |
| Male | 1,056 | 36.8 | 35.0 – 38.5 |
| Female | 1,816 | 63.2 | 61.5 – 65.0 |
| *Education* |  |  |  |
| High school and above | 220 | 7.7 | 6.7 – 8.6 |
| Primary/secondary school | 1,122 | 39.1 | 35.5 – 37.0 |
| Read and write only | 1,012 | 35.2 | 37.3 – 40.9 |
| Illiterate | 518 | 18.0 | 16.6 – 19.4 |
| *Marital status* |  |  |  |
| Married | 1,569 | 54.8 | 33.0 – 56.6 |
| Widowed | 1,225 | 42.8 | 41.0 – 44.6 |
| Separated, divorced, or single | 70 | 2.4 | 1.9 – 3.0 |
| *Living with spouse* |  |  |  |
| Yes | 876 | 30.5 | 28.8 – 32.2 |
| No | 1,994 | 69.5 | 67.8 – 71.1 |
| *Living with son/daughter* |  |  |  |
| Yes | 2,050 | 71.4 | 69.8 – 73.1 |
| No | 820 | 28.6 | 26.9 – 30.2 |
| *Living with grandchild* |  |  |  |
| Yes | 1,689 | 58.9 | 57.1 – 60.7 |
| No | 1,181 | 41.2 | 39.4 – 43.0 |
| *Living alone* |  |  |  |
| Yes | 273 | 9.5 | 8.4 – 10.6 |
| No | 2,597 | 90.5 | 89.4 – 91.6 |
| *Household head* |  |  |  |
| Yes | 1,493 | 52.1 | 50.2 – 53.9 |
| No | 1,374 | 47.9 | 46.1 – 49.7 |
| *Working status* |  |  |  |
| Yes | 1,160 | 40.4 | 38.6 – 42.2 |
| No | 1,713 | 59.6 | 57.8 – 61.4 |
| *Household size* |  |  |  |
| ≤ 4 persons | 1,481 | 51.5 | 49.7 – 53.4 |
| > 4 persons | 1,392 | 48.5 | 46.6 – 50.3 |
| *Area of residence* |  |  |  |
| Lowland | 866 | 30.1 | 28.5 – 31.8 |
| Highland | 1,430 | 49.8 | 49.1 – 51.6 |
| Mountainous | 577 | 20.1 | 18.6 – 21.6 |
| *Wealth quintile* |  |  |  |
| Richest | 603 | 21.0 | 19.5 – 22.5 |
| Richer | 639 | 22.2 | 20.7 – 23.8 |
| Middle | 637 | 22.2 | 20.7 – 23.7 |
| Poorer | 496 | 17.3 | 15.9 – 18.7 |
| Poorest | 498 | 17.3 | 16.0 – 18.7 |
| *National poverty line* |  |  |  |
| Above | 2,445 | 85.1 | 83.3 – 86.4 |
| Below | 428 | 14.9 | 13.6 – 16.2 |
| *Number of NCDs* |  |  |  |
| None | 1,662 | 58.0 | 56.1 – 59.8 |
| One | 841 | 29.3 | 27.7 – 31.0 |
| Two | 265 | 9.2 | 8.2 – 10.3 |
| Three or more | 100 | 3.5 | 2.8 – 4.2 |

**Table S2 – Percentages of older people who need some or complete support for at least one Activity of Daily Living (ADL), by socioeconomic group**

| Socioeconomic group |  | Basic ADLs | |  |  | Instrumental ADLs | |  |  | Intellectual ADLs | |  |
| --- | --- | --- | --- | --- | --- | --- | --- | --- | --- | --- | --- | --- |
|  | *%* | | *95%CI* | | *%* | | *95%CI* | | *%* | | *95%CI* | |
| *Age (yr)* |  | |  | |  | |  | |  | |  | |
| 60+ | 9.6 | | 8.6 – 10.7 | | 70.1 | | 68.4 – 71.7 | | 68.9 | | 67.2 – 70.6 | |
| 65+ | 11.7 | | 10.3 – 13.0 | | 76.0 | | 74.2 – 77.8 | | 74.1 | | 72.3 – 75.9 | |
| 70+ | 14.5 | | 12.8 – 16.2 | | 81.8 | | 80.0 – 83.7 | | 80.7 | | 78.8 – 82.6 | |
| 75+ | 18.8 | | 16.5 – 21.1 | | 87.1 | | 85.2 – 89.1 | | 85.8 | | 83.7 – 87.8 | |
| *Age group (yr)* |  | |  | |  | |  | |  | |  | |
| 60–69 | 3.0 | | 2.0 – 3.9 | | 53.9 | | 51.1 – 56.7 | | 52.6 | | 49.8 – 55.4 | |
| 70–79 | 8.1 | | 6.5 – 9.7 | | 75.6 | | 73.0 – 78.1 | | 75.4 | | 72.8 – 78.0 | |
| 80+ | 26.2 | | 22.6 – 29.7 | | 93.2 | | 91.2 – 95.3 | | 90.5 | | 88.1 – 92.9 | |
| *Sex* |  | |  | |  | |  | |  | |  | |
| Male | 7.5 | | 5.9 – 9.1 | | 76.1 | | 73.6 – 78.7 | | 49.8 | | 46.8 – 52.8 | |
| Female | 10.9 | | 9.5 – 12.3 | | 66.6 | | 64.4 – 68.8 | | 80.0 | | 78.2 – 81.9 | |
| *Education* |  | |  | |  | |  | |  | |  | |
| High school and above | 5.5 | | 2.4 – 8.5 | | 55.5 | | 48.8 – 62.1 | | 41.8 | | 35.3 – 48.4 | |
| Primary/secondary school | 5.2 | | 3.9 – 6.5 | | 62.9 | | 60.1 – 65.8 | | 49.9 | | 47.0 – 52.8 | |
| Read and write only | 9.3 | | 7.5 – 11.1 | | 72.6 | | 69.9 – 75.4 | | 80.4 | | 78.0 – 82.9 | |
| Illiterate | 21.8 | | 18.3 – 25.4 | | 86.7 | | 83.7 – 89.6 | | 98.8 | | 97.9 – 99.8 | |
| *Marital status* |  | |  | |  | |  | |  | |  | |
| Married | 5.7 | | 4.5 – 6.8 | | 65.7 | | 63.4 – 68.1 | | 58.5 | | 56.1 – 61.0 | |
| Widowed | 11.4 | | 3.8 – 19.1 | | 52.9 | | 40.9 – 64.9 | | 65.7 | | 54.3 – 77.1 | |
| Separated, divorced, or single | 14.7 | | 12.7 – 16.7 | | 76.6 | | 74.1 – 79.0 | | 82.5 | | 80.3 – 84.6 | |
| *Living with spouse* |  | |  | |  | |  | |  | |  | |
| Yes | 5.5 | | 4.0 – 7.0 | | 60.2 | | 56.9 – 63.4 | | 63.2 | | 60.0 – 66.4 | |
| No | 11.5 | | 10.1 – 12.9 | | 74.4 | | 72.5 – 76.3 | | 71.4 | | 69.4 – 73.4 | |
| *Living with son/daughter* |  | |  | |  | |  | |  | |  | |
| Yes | 7.8 | | 6.6 – 9.4 | | 58.8 | | 56.3 – 61.3 | | 67.8 | | 65.4 – 70.2 | |
| No | 11.4 | | 9.8 – 13.1 | | 82.0 | | 80.0 – 84.1 | | 70.0 | | 67.6 – 72.5 | |
| *Living with grandchild* |  | |  | |  | |  | |  | |  | |
| Yes | 11.8 | | 10.2 – 13.3 | | 81.0 | | 79.1 – 82.9 | | 72.8 | | 70.7 – 75.0 | |
| No | 6.6 | | 5.2 – 8.0 | | 54.4 | | 51.5 – 57.2 | | 63.3 | | 60.5 – 66.0 | |
| *Living alone* |  | |  | |  | |  | |  | |  | |
| Yes | 8.1 | | 4.8 – 11.3 | | 47.6 | | 41.7 – 53.6 | | 74.4 | | 69.2 – 79.6 | |
| No | 9.8 | | 8.7 – 11.0 | | 72.4 | | 70.7 – 74.1 | | 68.3 | | 66.5 – 70.1 | |
| *Household head* |  | |  | |  | |  | |  | |  | |
| Yes | 6.2 | | 5.0 – 7.5 | | 66.1 | | 63.7 – 68.5 | | 58.4 | | 55.9 – 60.9 | |
| No | 13.4 | | 11.6 – 15.2 | | 74.2 | | 71.9 – 76.6 | | 80.3 | | 78.2 – 82.4 | |
| *Working status* |  | |  | |  | |  | |  | |  | |
| Yes | 2.0 | | 1.2 – 2.8 | | 54.9 | | 52.1 – 57.8 | | 57.2 | | 54.4 – 60.1 | |
| No | 14.8 | | 13.1 – 16.5 | | 80.3 | | 78.4 – 82.2 | | 76.8 | | 74.8 – 78.8 | |
| *Household size* |  | |  | |  | |  | |  | |  | |
| ≤ 4 persons | 8.0 | | 6.6 – 9.3 | | 58.8 | | 56.3 – 61.3 | | 67.8 | | 65.4 – 70.2 | |
| > 4 persons | 11.4 | | 9.8 – 13.1 | | 82.0 | | 80.0 – 84.1 | | 70.0 | | 67.6 – 72.5 | |
| *Area of residence* |  | |  | |  | |  | |  | |  | |
| Lowland | 11.2 | | 9.1 – 13.3 | | 65.1 | | 62.0 – 68.3 | | 74.0 | | 71.1 – 77.0 | |
| Highland | 8.7 | | 7.2 – 10.1 | | 70.1 | | 67.8 – 72.5 | | 70.8 | | 68.5 – 73.2 | |
| Mountainous | 9.7 | | 7.3 – 12.1 | | 77.3 | | 73.9 – 80.7 | | 56.3 | | 52.3 – 60.4 | |
| *Wealth quintile* |  | |  | |  | |  | |  | |  | |
| Richest | 9.5 | | 7.1 – 11.8 | | 68.7 | | 64.9 – 72.4 | | 59.4 | | 55.4 – 63.3 | |
| Richer | 10.0 | | 7.7 – 12.4 | | 71.7 | | 68.2 – 75.2 | | 65.6 | | 61.9 – 69.3 | |
| Middle | 8.8 | | 6.6 – 11.0 | | 71.7 | | 68.2 – 75.3 | | 68.8 | | 65.2 – 72.4 | |
| Poorer | 9.7 | | 7.1 – 12.3 | | 69.6 | | 65.5 – 73.6 | | 73.6 | | 69.7 – 77.5 | |
| Poorest | 10.4 | | 7.8 – 13.1 | | 68.1 | | 64.0 – 72.2 | | 80.1 | | 76.6 – 83.6 | |
| *National poverty line* |  | |  | |  | |  | |  | |  | |
| Above | 8.9 | | 7.8 – 10.0 | | 69.3 | | 67.5 – 71.2 | | 66.7 | | 64.8 – 68.6 | |
| Below | 14.0 | | 10.7 – 17.3 | | 74.3 | | 70.1 – 78.5 | | 81.3 | | 77.6 – 85.0 | |
